# Estimation of Genome Size in the Endemic Species *Reseda pentagyna* and the Locally Rare Species *Reseda lutea* Using comparative Analyses of Flow Cytometry and K-Mer Approaches

**DOI:** 10.3390/plants10071362

**Published:** 2021-07-03

**Authors:** Fahad Al-Qurainy, Abdel-Rhman Z. Gaafar, Salim Khan, Mohammad Nadeem, Aref M. Alshameri, Mohamed Tarroum, Saleh Alansi, Naser B. Almarri, Norah S. Alfarraj

**Affiliations:** 1 Department of Botany and Microbiology, College of Science bldg5, King Saud University, Riyadh 11451, Saudi Arabia; falqurainy@ksu.edu.sa (F.A.-Q.); skhan2@ksu.edu.sa (S.K.); mnadeem@ksu.edu.sa (M.N.); aalshameri@ksu.edu.sa (A.M.A.); mtarroum@ksu.edu.sa (M.T.); salansi@ksu.edu.sa (S.A.); 438203416@student.ksu.edu.sa (N.S.A.); 2 Ministry of Environment, Water and Agriculture, Riyadh 11195, Saudi Arabia; almarri@moa.gov.sa

**Keywords:** flow cytometry, endemic, ploidy, rare, *Reseda*, genome size, k-mer

## Abstract

Genome size is one of the fundamental cytogenetic features of a species, which is critical for the design and initiation of any genome sequencing projects and can provide essential insights in studying taxonomy, cytogenetics, phylogenesis, and evolutionary studies. However, this key cytogenetic information is almost lacking in the endemic species *Reseda pentagyna* and the locally rare species *Reseda lutea* in Saudi Arabia. Therefore, genome size was analyzed by propidium iodide PI flow cytometry and compared to k-mer analysis methods. The standard method for genome size measures (flow cytometry) estimated the genome size of *R. lutea* and *R. pentagyna* with nuclei isolation MB01 buffer were found to be 1.91 ± 0.02 and 2.09 ± 0.03 pg/2 °C, respectively, which corresponded approximately to a haploid genome size of 934 and 1.022 Mbp, respectively. For validation, K-mer analysis was performed on both species’ Illumina paired-end sequencing data from both species. Five k-mer analysis approaches were examined for biocomputational estimation of genome size: A general formula and four well-known programs (CovEST, Kmergenie, FindGSE, and GenomeScope). The parameter preferences had a significant impact on GenomeScope and Kmergenie estimates. While the general formula estimations did not differ considerably, with an average genome size of 867.7 and 896. Mbp. The differences across flow cytometry and biocomputational predictions may be due to the high repeat content, particularly long repetitive regions in both genomes, 71% and 57%, which interfered with k-mer analysis. GenomeScope allowed quantification of high heterozygosity levels (1.04 and 1.37%) of *R. lutea* and *R. pentagyna* genomes, respectively. Based on our observations, R. lutea may have a tetraploid genome or higher. Our results revealed fundamental cytogenetic information for *R. lutea* and *R. pentagyna*, which should be used in future taxonomic studies and whole-genome sequencing.

## 1. Introduction

The development of advanced genomic technologies, and the subsequent storm of data from next-generation sequencing (NGS), has been a great asset to genomic research. However, many fundamental issues concerning genomes remain mostly unresolved. One such issue is the largely unexplored amount of DNA (C-value) in most of the higher clades of life. The amount of DNA (C-value) in the haploid gametic nucleus is referred to as genome size [1], which is often quantified in picograms (pg) or megabase pairs (1 pg = 978 Mbp) [2] and is typically broadly constant within an organism [3,4]. Besides external characteristics, genome size is a key value for research on taxonomy, ecology, and evolution [5,6]. Variations significant enough to differentiate a population into distinct species may still be difficult to discern employing classic morphological or DNA sequence; nevertheless, such variations may become more obvious when genome size is investigated along with other proofs [7,8]. Moreover, a precise calculation of genome size is a prerequisite in the age of high-throughput sequencing technologies for sequencing projects [7], since it influences the budget plan for anticipated sequencing depths and offers an approximate figure for estimating genome assembly completeness.

As a result, there is a great demand for reliable and easy-to-use methods for calculating genome sizes throughout a wide range of eukaryotic taxa [9]. There are two main methods for calculating genome size: Laboratory and computational. The Feulgen microdensitometer and flow cytometry are fairly tested and often used laboratory approaches [10]. Flow cytometry is a low-cost, relatively reliable, and quick laboratory technique for estimating plant genome size. It is an appealing alternative to microspectrophotometry in that it involves the calculation of DNA quantities based on the staining of undamaged nuclei with a fluorochrome that quantitatively adheres to the DNA. Moreover, for the analysis, only a small amount of tissue is needed, which is important in the case of valuable and/or protected specimens [10,11]. These methods, however, rely on living, adequately fixed, or frozen tissues with substantially intact cells, thereby limiting research to lifeforms that can be cultivated in the lab or easily obtained in the field and transferred to the lab [12]. Furthermore, considering that the significant amounts of phenolic compounds can create stoichiometric errors, the flow cytometry approach must be tailored to each plant species.

Meanwhile, with the explosive growth of next-generation sequencing technology, a computational technique arose through k-mer (distinct subsequences of a given length, k, derived from a longer DNA sequence) approaches. A k-mer frequency distribution could be generated by plotting the coverage distribution over all k-mers in a sequence. This k-mer distribution should resemble a Poisson distribution when the created k-mers from genomic sequencing reads possess minuscule amounts of sequence defects (repetitions, sequencing errors, or coverage bias). The distribution peak will be centered on the average sequencing depth for the genome [13]. K-mer based genome size estimates were accurately employed in many genome projects due to their feasibility and rationale [14,15,16,17]. Researchers can utilize many available programs to estimate genome size using sequencing data as well as the popular equation, i.e., the quotient of the k-mers total number and the peak frequency distribution. However, the accuracy and efficiency of these strategies have not been thoroughly investigated.

The Resedaceae is a relatively small family with only six genera (i.e., *Reseda*, *Randonia*, *Sesamoides*, *Oligomeris*, *Ochradenus*, and *Caylusea*) and about 85 species [18]. Genus *Reseda* contains approximately 65 species throughout the world, mostly restricted to the Mediterranean basin. Several of its species flourish on soils under arid environments, while others are ruderal weeds and only a few are available in high mountains [19]. Pharmacological studies of various *Reseda* species showed antimicrobial [20], anti-inflammatory [21], and antioxidant [22] activities. In Saudi Arabia, seven species of the genus *Reseda* were recorded, viz. *R. alba*, *R. arabica*, *R. aucheri*, *R. lutea*, *R. muricata*, *R. pentagyna,* and *R. sphenocleoides* [23]. Among these, *R. pentagyna* is endemic and native to Saudi Arabia and has been observed in northeastern region in Tabuk, Wadi Sawawin, and Northern Hijaz Mountain range [24]. *R. pentagyna* is an annual sparsely branched herbaceous plant well adapted to hard sand and low rocky hills with stems erect up to 30 cm, distinguished from the other *Reseda* species via its five to six-toothed capsule [25]. While *R. lutea* L. is locally rare and restricted only to a single gathering in the mountainous region of Abha, Saudi Arabia [26]. *R. lutea* L. is a deep-rooted biennial or perennial herbaceous plant that can grow up to 80 cm high and well adapted to fallow fields, rocky slopes, and roadsides. It is distributed and spread throughout many temperate zones of the world [27]. This study aimed to determine the genome size focusing on the endemic species *R. pentagyna* and the locally rare species *R. lutea* both experimentally using flow cytometry and computationally using the k-mer approach through a combination of short-read sequencing with bioinformatics tools.

## 2. Material and Methods

### 2.1. Plant Material

Seeds from adult plants of both the endemic species *R. pentagyna* and the rare species *R. lutea* were collected from Abha, Saudi Arabia, for in vitro plant propagation. The identification was confirmed through morphological features coupled with the assistance of Flora of Saudi Arabia [28] and protologue [29], and a voucher specimen (SBSN00015 and SBSN00016) was deposited at the Seed Bank Herbarium, College of Sciences, King Saud University, KSA. The intact seeds were surface-sterilized with 0.3% sodium hypochlorite for 2 to 3 min, then washed 3 to 4 times with double-sterilized water. The seeds were germinated on 2% agar then inoculated on Murashige and Skoog (MS) medium [30].

### 2.2. Genomic DNA Extraction

**Figure 1:**
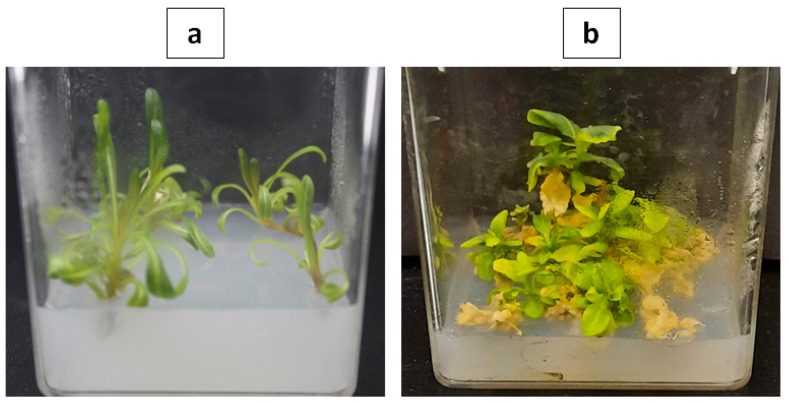
Shoots raised on MS medium (**a**) *Reseda lutea*; (**b**) *Reseda pentagyna*.

A leaf sample from germinated seeds was detached from the medium and directly used for DNA isolation (Figure 1). Total genomic DNA was isolated from *R. lutea* and *R. pentagyna* leaves using the DNeasy Plant Mini Kit (Qiagen, Valencia, CA, USA) according to the manufacturer’s instructions. The NanoDrop2000 spectrophotometer was used to evaluate the purity and amount of DNA (Thermo Fisher Scientific, Waltham, MA, USA). DNA integrity was determined using a 1% (*w/v*) agarose gel electrophoresis. The nuclear ITS region (internal transcribed spacer sequences) was amplified on an AB Veriti 96 well Thermal cycler (Applied Biosystems, Waltham, MA, USA) using PuReTaq Ready-To-Go PCR Beads (GE Healthcare, Little Chalfont, Buckinghamshire, UK). Universal ITS primers were used for amplification and cycle sequencing (ITS1 and ITS4 [31,32]) using the following conditions: Initial denaturation at 94 °C for 5 min, 25 cycles of denaturation for 30 s at 94 °C, annealing at 48 °C for 30 s, extension at 72 °C for 1 min, and a final extension at 72 °C for 7 min. PCR reactions were examined on a 1.2% (*w/v*) agarose gel to confirm the concentration and size of the PCR products. Following standard procedures, Macrogen Inc. (Geumchun-gu, Seoul, South Korea) used a 96-capillary ABI 3730xl DNA analyzer (Applied Biosystems, Foster City, CA, USA) to sequence the amplicons bidirectionally.

### 2.3. Molecular Identification

**Figure 2:**
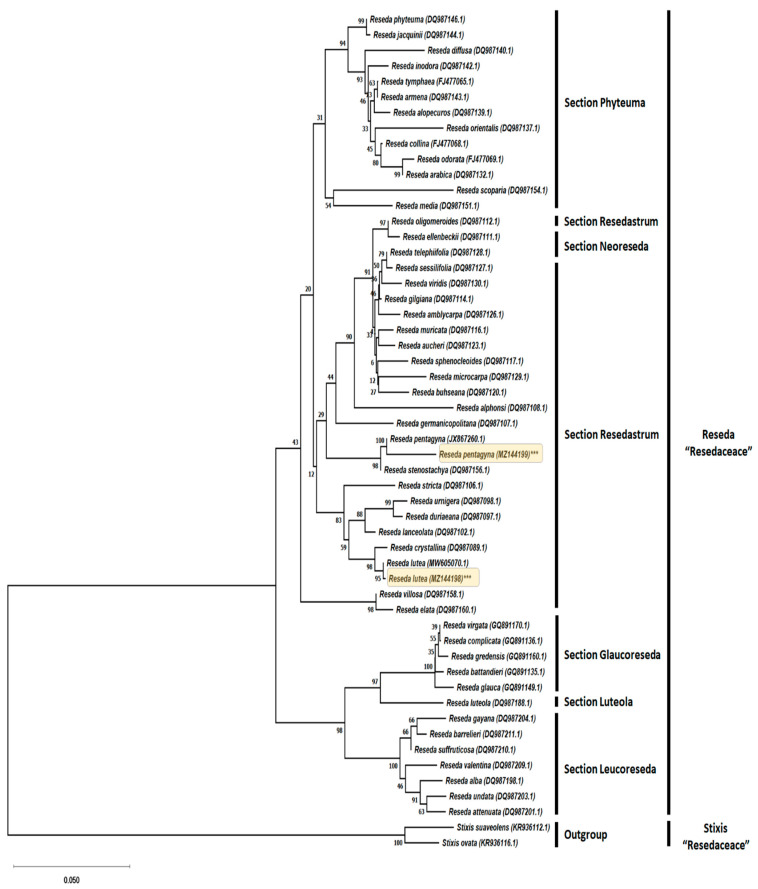
Molecular identification of *Reseda lutea* and *Reseda pentagyna* (highlighted) was conducted via evolutionary analyses in MEGA X and inferred from the analysis of internal transcribed spacer (ITS) sequence using the Neighbor-Joining algorithm. Next to the branches is the percentage of replicate trees around which the linked taxa grouped with each other in the bootstrap test (2000 replicates). The Kimura 2-parameter model was used to calculate the evolutionary distances (NCBI accession numbers between brackets).

For molecular identification and phylogenetic assessment, ITS sequences from 50 related *Reseda* species (including representatives from each of the six sections, namely Resedastrum, Phyteuma, Neoreseda, Luteola, Leucoreseda, and Glaucoreseda [18]) were acquired from GenBank (Figure 2). Sequences of two species from the genus *Stixis* (Resedaceae*)* were selected and retrieved from GenBank as the outgroup in the phylogenetic analyses (Figure 2). All analyses were implemented in MEGA X [33]. Sequence alignments were performed using Clustal W within the MEGA X windows interface, with manual adjustments. The Neighbor-Joining (NJ) method was utilized for phylogenetic analysis, and the model test was employed to identify the best-fit model for the NJ analysis (Kimura 2-parameter model with a discontinuous Gamma distribution K2 + G). The NJ method was selected for the construction of the phylogenetic tree because it has demonstrated advantages over distance and parsimony approaches to analyze the process of sequence evolution [34]. To obtain statistical support for every internal and external branch, a bootstrap test with 2000 replication was run concurrently for all analyses.

### 2.4. Flow Cytometric Genome Size

The young leaves from multiple shoots raised on MS media were used for the extraction of nuclei. Dr. Jaroslav Dolezel (Laboratory of Molecular Cytogenetics and Cytometry, Institute of Experimental Botany, Sokolorakrá 6, Olomouc, Czech Republic) kindly offered the seeds of external reference *Solanum lycopersicum* cv. Stupicke (2C = 1.96 pg) [35]. MB01 buffer [36] was used for the estimation of 2C DNA content of *Reseda lutea* and *Reseda pentagyna* (2.5 mM Na2EDTA; 20 mM MOPS; 0.2% (*v/v*) Triton X-100; 80 mM KCl; 0.7 mM Spermine tetrahydrochloride; 20 mM NaCl; pH 7.4). In addition, antioxidants including 1% PVP and 0.5% β-mercaptoethanol were freshly prepared and added for extraction of pure nuclei.

All experiment steps of nuclei extraction were performed on ice (4 °C). The young leaves (30 mg) were chopped with a sharp razor blade into 0.3–0.6 mm size in a petri dish containing ice-cold 500 µL MB01 nuclei isolation buffer. The suspension was mixed by pipetting and filtered through a 20 µM double nylon mesh. After filtration, the nuclei suspension was stained for 10 min with 50 µg/mL of PI (Propidium iodide, Sigma, St. Louis, MO, USA) under dark refrigeration, and the samples were stored on ice prior to analysis.

The fluorescence of a minimum of 5000 propidium iodide-stained nuclei was estimated using a flow cytometer Muse cell analyzer (Merck Millipore, Burlington, MA, USA). The flow rate of the capillary was set at 0.12 µL/s, which is very low. Propidium iodide was measured at 585 nm to read the 2C nuclei DNA content of the sample. The obtained histograms were computerized by Muse cell analyzer software package (Muse 1.8 analyses, Burlington, MA, USA). The sample 2C DNA content was calculated according to the formula [37]:

(1) $$2C\;\mathrm{DNA}\;\mathrm{content}\;\mathrm{of}\;\mathrm{sample}=\frac{\left(\mathrm{Fluorescence}\;\mathrm{mean}\;\mathrm{intensity}\;\mathrm{of}\;\mathrm{sample}\right)}{\left(\mathrm{Fluorescence}\;\mathrm{mean}\;\mathrm{intensity}\;\mathrm{of}\;\mathrm{standard}\right)}\times 2C\;\mathrm{DNA}\;\mathrm{content}\;\mathrm{of}\;\mathrm{standard}$$

The number of base pairs per haploid genome was determined using the formula 1 pg DNA = 978 Mbp [2,38]. Three replicate measurements were taken for each plant species independently. The fluorescence histograms were resolved into G0/G1 (2C), S, and G2/M (4C) cell-cycle compartments. The fluorescence mean intensity was taken for the calculation of the 2C DNA content of *Reseda* species. To improve accuracy, the genome size was determined for each sample as the mean of two technical and three biological replicates, enabling the standard error to be calculated.

### 2.5. Whole-Genome Sequencing and Filtering Contaminated Reads

Macrogen Inc. prepared the DNA libraries for genome sequencing (S. Korea). Using the TruSeq Nano DNA kit, a paired-end 350 bp insert size library was created for the two species (Illumina, San Diego, California , USA). The libraries were then sequenced using 2 × 151 bp paired-end sequencing on the Illumina NovaSeq6000 platform using standard Illumina operating protocol, yielding a minimum of 90 Gb of raw data. The run’s primary data processing was completed with the manufacturer’s program Real-Time Analysis (RTA 1.18.66.3), followed by the construction of FASTQ sequence files with the Illumina tool bcl2fastq. The raw sequencing reads were deposited in the GenBank database under the BioProject accession PRJNA733338. FastQC v0.11.9 [39] was used to visually examine the raw read quality. Trimmomatic (v0.38) [40] was used to delete the remaining adapter sequences, leading and trailing nucleotides with a Phred score of less than 25, and reads less than 50 bp. SOAPec v2.03 [41] was used to fix the errors in filtered reads. FastUniq v1.1 [42] was used to remove duplicated read pairs. All reads were filtered of potential contaminants by mapping via the BBDuk module (BBMap v38.9 [43]) against a contamination database that included chloroplast, mitochondrial, bacterial, and viral sequences, etc., detected with FastQ Screen [44] keeping only unmapped reads and subsequently assessed again using FastQC.

### 2.6. K-Mer Based Genome Size

Even though the genome size can be calculated by tallying the k-mer frequency of the read data, the k-mer must be high enough to differentiate most of the genome. The optimal k-mer length for genome size estimation has not been extensively tested. The k-mer value varies amongst investigations, whereas values between 17 and 35 are prominent [45,46]. At least 17 are commonly employed in most eukaryotic genomes to prevent palindromic sequences and the effect of excessively repetitive DNA sequences. For analysis, first the frequency distribution of three k-mers (i.e., 21, 31, and 41) was generated using Jellyfish v2.3.0 [47]. Second, four k-mer analysis-based methods were evaluated for computational genome size estimation, including the most recent dedicated tools (Kmergenie v1.7 [48], GenomeScope v1 [49], FindGSE v1.94 [50], and CovEST-repeat [51]) and the commonly used formula for the calculations of genome size sourced from the equation (M = N × (L – K + 1)/L) proposed by the M.S. Waterman group, where (M) the reads k-mer frequency peak is associated with (N) the actual sequencing depth, (K) kmer length, and (L) read length [13,52,53]. Third, the ploidy structure was estimated with Smudgeplot v0.2.3 [54]. Finally, GenomeScope v1 was run using k-mer length (k = 21) and analyzed the histograms to estimate the complexity of the genome (heterozygosity and repeats) with maximal k-mer coverage = − 1.

## 3. Result and Discussions

### 3.1. Molecular Identification

Internal transcribed spacer ITS sequences of nuclear ribosomal DNA have received a lot of attention over the last two decades, not only because of their effectiveness in performing plant phylogeny at a lower taxonomic level, but also because they are regarded as far more reliable markers available for plant DNA barcoding. Due to the highly intriguing morphological similarities reported across *Reseda* species [28,29], molecular identification and phylogenetic analysis with ITS were implemented to determine the species designation of *R. lutea* and *R. pentagyna*.

To validate the morphology-based taxonomic identification of *R. pentagyna* and *R. lutea*, the ITS region was sequenced and aligned to 50 *Reseda* species with ITS sequences currently available at NCBI (including the ones for both *R. lutea* and *R. pentagyna*). The combined length of ITS region for the two plants comprised 699 and 707 nucleotides, respectively. A BLAST screening of *R. pentagyna*’s ITS query sequence revealed the highest sequence identity and similarity to previously published *R. pentagyna* ITS sequences JX867260.1 97.95% and similarly *R. lutea* 99.86% for itself KR936125.1.

The Neighbor-Joining algorithm was used to infer the evolutionary phylogram tree with the lowest BIC (Bayesian Information Criterion) score of 9855.053 based on the Kimura 2-parameter model to estimate a matrix of pairwise distances. The evolutionary rate differences between sites were modeled using a discrete Gamma distribution (5 categories). The tree is depicted to scale, and branch lengths are calculated by counting the number of substitutions for each site. The tree was rooted with the help of *Stixis suaveolens* (KR936112.1) and *Stixis ovata* (KR936116.1) as an outgroup. Bootstrap supports (%) with a value greater than 50% are displayed above branches.

The Neighbor-Joining tree derived from the analysis of ITS sequences is in line with previous phylogenetic analyses and revealed grouping of *Reseda* species consistent with established taxonomic sections of the genus, *R. pentagyna* showed proximity with *R. stenostachya* (98% bootstrap support), while *R. lutea* showed proximity with *R. crystallina* (99% bootstrap support) nested within the clade of section Resedastrum (Figure 2). The research concluded that *Reseda* species were grouped and consistent with preexisting taxonomic sections [18]. As a result, our ITS analysis validated the taxonomic identification and classification of the examined plants based on morphology.

### 3.2. C-Value Determination via Flow cytometry

Due to the development of flow cytometry, the study of genome size and its significance has dramatically increased in recent years not just as a taxonomic marker, but also for assessing how it corresponds to environmental, ecological, and phenotypic variables [55,56,57,58]. Furthermore, before determining the nucleotide sequence of a plant’s DNA, it is necessary to understand how large the genome is [59]. According to a large-scale analysis of plant genome sizes, large genomes are less resistant to environmental pressures like drought or pollution, and are less capable of adjusting, making them more vulnerable to extinction [60,61]. Consequently, the genome size evolution heads toward small genomes [59]. Therefore, knowledge of the genome size of the two species of *Reseda* under study could be used for the prediction of the threat of extinction particularly the rare species *R. lutea* [60].

**Figure 3:**
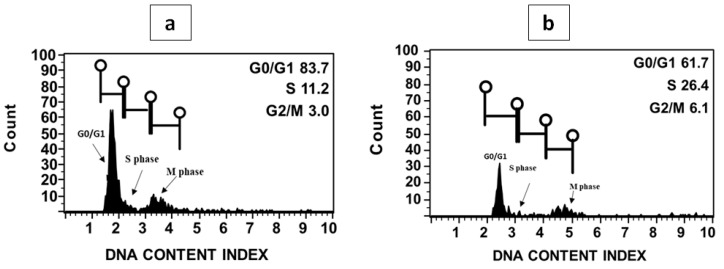
Histogram of fluorescence intensity for genome size assessments in *R. lutea* (**a**) and *R. pentagyna* (**b**) nuclei stained with propidium iodide prepared from shoot tissues. The two major phases of the cell cycle (interphase G0, G1, S, G2 and the mitotic phase M).

Preliminary testing revealed the success of flow cytometry analyses with both *Reseda* species forming peaks in the histograms. The 2C peaks in the histograms for fresh plant materials were suitable for genome size estimation (Figure 3). The nuclear DNA content of the two species of *Reseda* was evaluated by flow cytometry using tomato (2C = 1.96 pg) as an external reference standard, which was later determined to be the most appropriate standard for *Reseda* samples due to their proximate DNA content. The genome size for *R. lutea* and *R. pentagyna* showed a narrow range and was estimated to be 1.91 ± 0.02 and 2.09 ± 0.03, respectively (Table 1). Our estimations for *R. lutea* and *R. pentagyna* constitute one of the highest values so far for this genus *Reseda* (0.92–2.86 pg/2C). For *R. lutea*, whose genome size had previously been assessed, there was a clear agreement with earlier findings (Table 2). The slight difference in DNA content could occur due to the type of laser lamp equipped in the flow cytometer [62]. According to Soltis et al. [63] classification, both *Reseda* species genomes belong to the category of plants with a smaller genome. The genome of *R. pentagyna* is around the same size as that of *R. lutea*, an octoploid species [18]. Furthermore, its genome is nearly twice as large as that of *R. suffruticosa*, which possesses a tetraploid genome [18,64].

### 3.3. Whole-Genome Sequencing

**Figure 4:**
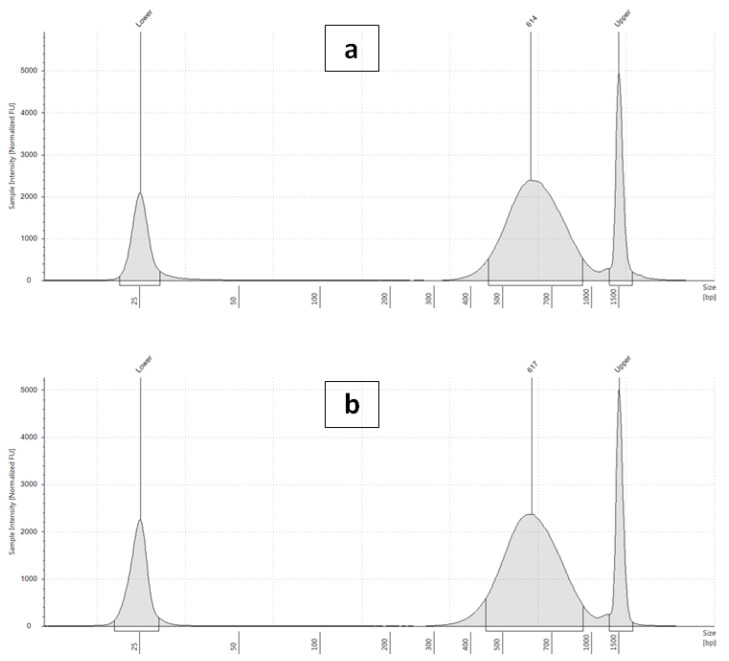
Quality check electropherogram of post-library construction on the Agilent 2200 TapeStation (**a**) *Reseda lutea* and (**b**) *Reseda pentagyna*.

**Figure 5:**
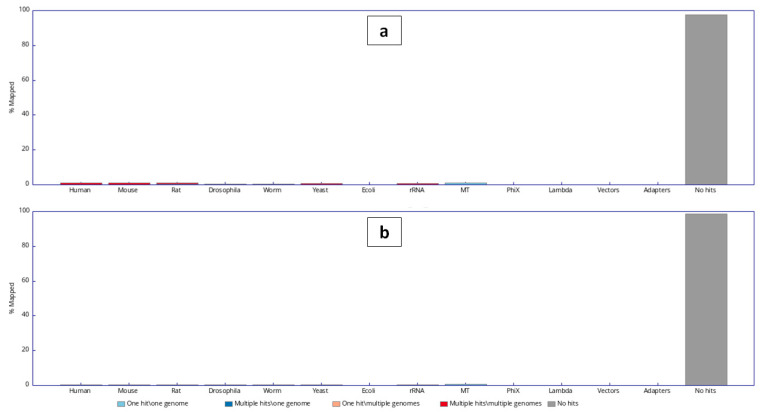
The plot shows the frequency distribution of the contaminating taxa for (**a**) *Reseda lutea* and (**b**) *Reseda pentagyna*.

**Figure 6:**
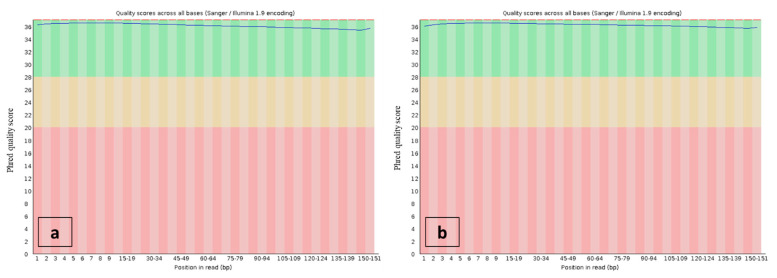
Phred quality scores throughout the read bases—dataset ready for genome size estimation—*Reseda lutea* (**a**) and *Reseda pentagyna* (**b**).

The development of improved sequencing technology capable of producing considerable amounts of sequence data at a low cost, combined with enhanced assembling procedures, has expanded both model and non-model plants genome sequencing [66]. The paired-end 350 bp insert size libraries (Figure 4) of *R. lutea* and *R. pentagyna* were sequenced using the HiSeq 2500 Illumina sequencing platform, which produced 358.2 and 352.4 million pairs of 151bp reads, accounting for a total of 108.2 Gb and 106.4 Gb of sequence, respectively. Based on the flow cytometry estimates of genome size, the sequence data represented more than 100× coverage of both genomes (Table 3). Tools for estimating genome size employing k-mer distributions perform much better whenever the average coverage is higher than 10× [67]. Quality filtering (removing bases with a Phred score of less than 25 and reads shorter than 50 bp) did not significantly decrease the dataset. Approximately 0.6–1.5% of the reads identified by FastQ Screen (Figure 5) as contaminants (chloroplast, mitochondrial, bacterial, and viral sequences, etc.), which in turn were used to map the clean reads with bbduk2, leaving between 637.9 Mbp and 632.3 Mbp unmapped reads for further processing (Table 3 and Figure 6). After the quality filtering was established, the raw data mean read length was 148bp.

### 3.4. K-Mer Based Genome Size and Complexity

Accurate genome size measurement is crucial for genome research projects [1], and it provides data for analyzing variation in genome size over a wide taxonomic group [68]. Nevertheless, calculating genome size effectively with flow cytometry demands the elimination of potential erroneous sources [12,69,70]. Flow cytometry analysis may exaggerate the measured values due to the impact of various plant metabolites on stain binding. Consequently, k-mer analysis was carried out to validate the flow cytometry findings. Although estimates based on k-mer analysis may vary depending on the program’s parameter choices, the quality of the sequencing data may also hold a role. Hence, four methods were investigated for computational genome size prediction using k-mer analysis, including the most notable trusted programs (CovEST-repeat, kmergenie, GenomeScope, and FindGSE) and the widely used equation for genome size calculations sourced from the formulas proposed by M.S. Waterman group. The GenomeScope authors suggested k-mer 21 as an acceptable compromise between both computation accuracy and speed [49], while k-mers ranging from 17–27 have been employed in other research [45,50,71]. In this study, all k-mer evaluations were executed with k values ranging from 21–41 to ensure that the k length had no effect on the estimations. The impacts of k-mer size (21-mer, 31-mer, and 41-mer) and raw vs. quality processed data were explored for each program (Table 4). The differences between raw and quality processed datasets were minor and skewed in favor of processed data.

According to our findings, the behavior of kmergenie and GenomeSope performance was drastically affected by increasing k-mer. The GenomeSope genome size estimates in processed data varied from 591 Mbp to 796 Mbp in *R. lutea* and from 524 Mbp to 748 Mbp in *R. pentagyna*. A closer examination of the kmergenie results revealed that the predicted genome was roughly half the output expected for both species’ haploid genomes, resulting in an underestimated genome size. This was also demonstrated in investigations with cane toad [72], vanilla [73], and Pacific oyster [49,74], where k-mer-based GenomeScope estimations of genome sizes were barely half of those derived by flow cytometry and far smaller than those achieved after genome assembly. The discrepancy demonstrates that these strategies might be unreliable in some instances. The genome size estimates from the other k-mer methods were generally slightly low compared to flow cytometry estimates, but different from CovEST “repeat” estimates, which were higher on average than the size suggested by the flow cytometry measurements. Such a basic pattern was also detected while matching whole-genome assemblies to flow cytometry and Feulgen staining [75].

GSE predicted *R. lutea* genome size of average 980.7 ± 101.2Mbp while 886.7 ± 96.4 Mbp in *R. pentagyna*. For both genomes with the General Formula prediction, the effects of using different kmer sizes were small (<0.016 Gbps). With this Formula, the genome size estimates for *R. lutea* and *R. pentagyna* of 907 ± 16.5 Mbp and 896.3 ± 26.6 Mbp. In general, the haploid genome size estimation of *R. lutea* based on k-mer distributions of the Illumina sequence reads ranged from 447.7Mbp (kmergenie), over 680 Mbp (GenomeScope), over 860 Mbp (General Formula), to over 950 Mbp (FindGSE) while *R. pentagyna* ranged from 552.3Mbp (kmergenie), over 630.3 Mbp (GenomeScope) to around 900 Mbp (FindGSE, General Formula) (Table 4).

**Figure 7:**
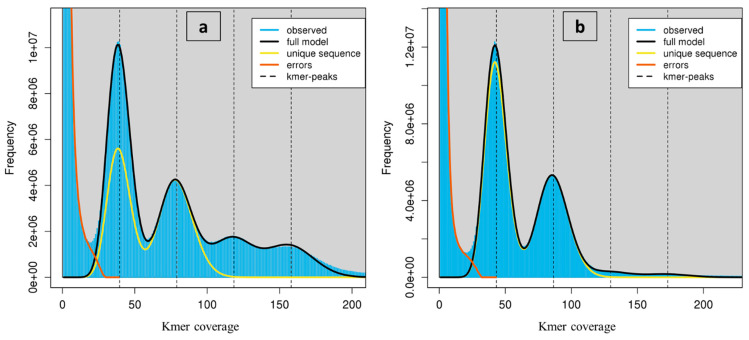
K-mer profile (*k* = 41) spectrum analysis to estimate genome size in *Reseda lutea* (**a**) and *Reseda pentagyna* (**b**) generated from sequence data using GenomeScope V1. The high peak at quite low depths, induced by sequencing errors, has been trimmed to empower visualization.

The k-mers depth distribution histograms (Figure 7) revealed a unique bimodal profile in both species with a high peak around 40× coverage and a shorter peak around 80×. This could be evidence of a highly heterogeneous genome [49]. Additionally, GenomeScope estimated that all genomes consisted of high repetitive sequences. Values of lower k-mers yielded much lower genome size estimates than suggested by flow cytometry, while larger k values produced estimates that were more consistent. The C-value determined by the General Formula k-mer analysis average value for *R. lutea* was 1.78 pg/2C, which is 0.13 pg lower than the C-value determined by flow cytometry. The proportion of repetitive sequences was determined to be approximately 71.26% based on the distribution of k-mers, while heterozygosity was approximately 1.04% (Table 5). The proportion of repetitive sequences and heterozygosity in *R. pentagyna* were approximately 56.77% and 1.37%, respectively. The C-value based on k-mer analysis was 1.84 pg/2C, which is 0.25 pg lower than that predicted from flow cytometry (Table 1). Similar inconsistencies have been documented for *Arabidopsis thaliana*, as well as European eels, and were attributed to chemical compounds interference in stoichiometric DNA content estimations in flow cytometry analysis [50,76].

However, the observed slight difference in genome size estimated for *R. lutea* and *R. pentagyna* when determined through using k-mer and flow cytometry methods could be attributable to the comparatively low sequencing depth as well as the relatively significant proportion of complex or long repetitive elements (>short reads) in these species’ nuclear genomes (Table 5) that were not recovered in the sequencing [77]. According to Kidwell [78], there is a close association between repetitive DNA sequences and genome size, and the link was demonstrated by Li et al. [79]. Once they account for a high fraction of the genome, repetitive elements are known to restrict genome size estimates downwards [80].

In maize, repeats account for approximately 80% [81] of the genome, with a sophisticated structure that complicates whole-genome sequencing [82]. These constraints could be quickly overcome with further participation of deep sequencing from third-generation sequencing technology. Additionally, the substantial genome size estimated for *R. lutea* and *R. pentagyna* (≈1 Gbp) indicates that constructing a high-quality (i.e., chromosomal level) genome will most likely require a combination of short and long reads (i.e., ONT, PacBio). Long reads with lengths of ~10–20 kbp [83] can allow clarification of repetitive genomic zones, while short reads, in turn, increase assembly accuracy since their error rate is relatively lower than long reads ones [84,85].

The kmer length had a significant impact on predicted genome size in both species (*p*-value >0.01—one-way ANOVA).

### 3.5. Ploidy Level Estimation

Detailed bibliographic research on the documented basic chromosome number and ploidy levels of the examined taxa was performed to determine the DNA ploidy level. In terms of chromosomal numbers and ploidy level (mostly from the following online databases and bibliography: Plant DNA C-values Database [86], Chromosome Counts Database (CCDB) [87], and Index of Plant Chromosome Numbers [88]).

In these studies, the basic chromosome number was proposed to be (× = 6) within the Resedastrum section (Table 2) with two ploidy levels (terta-, octoploid) [64,89], and species possessing chromosome counts *n* = 24 or more were proposed to have evolved from interspecific hybridization and the generation of reproductive plants through hybrid genome doubling [65]. Previously reported chromosome counts for *R. lutea* have been inconsistent [90] with most reports determining its chromosome number to be 2n = 48 [25,64] whereas few studies identified the chromosome numbers to be 24 [25]. Considering the documented chromosomal counts in *R. lutea* and its unavailability in *R. pentagyna* and depending on the comparable C value among both species, we hypothesize that these species possess the same number of chromosomes, 48.

**Figure 8:**
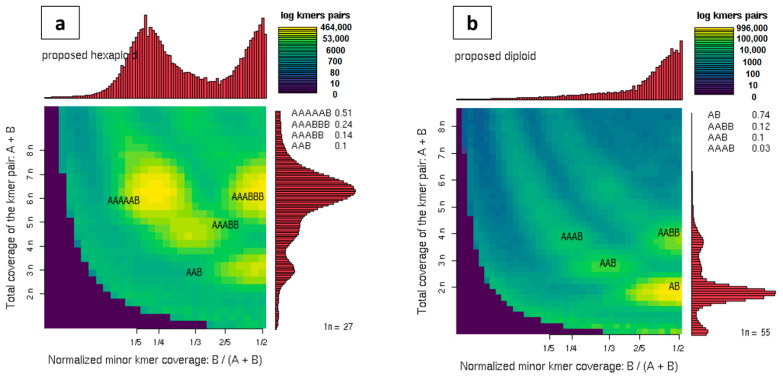
Two-dimensional heat maps were constructed to depict the prediction of ploidy from clean reads using Smudgeplot (k = 21). (**a**) *Reseda lutea* and (**b**) *Reseda pentagyna*. The color intensity corresponds to the approximate amount of k-mers per bin, ranging from purple (weak) to yellow (strong). Estimated ploidies are shown in the upper left corner of each graph, with the likelihood of various ploidies shown on the right.

Moreover, the wide range of DNA content between species (0.92–2.86 pg/2C) in the genus *Reseda* usually supports changes in ploidy, hence Illumina reads were used to assess the ploidy level via Smudgeplot, which uses the ratio of heterozygous k-mer pairs to estimate ploidy. Analysis of *R. lutea* sequence data provided hints that a polyploid genome, from analysis with a k-mer size of 21 with the most abundant k-mer pairs, is the hexaploid heterozygous (AAAAAB) form of *R. lutea* (Figure 8). To our knowledge, this is the first report of a hexaploid form of *R. lutea*. The probability of *R. lutea* possessing a polyploid genome has been implied based on the genome size expansion and the increase in the basic chromosome numbers.

Meanwhile, *R. pentagyna* Smudgeplot analysis supported a diploid heterozygous genome (AB) and not polyploidy, which may be the result of the occurrence of a strict uncommon autopolyploid phenomenon that has been revealed in some species [91] and the analysis tool could not interpret. Smudgeplot is designed to predict high heterozygous species and therefore fails to interpret a totally homozygous polyploid genome [54]. However, more cytological studies should be carried out to confirm the chromosome number and verify the ploidy type. Furthermore, because there is a good association between DNA content and ploidy within a species, population-size studies using flow cytometry could be undertaken in the future to differentiate ploidy levels within a species.

## 4. Conclusions

The significance of the genome size trait is self-evident, as it not only determines plant community configurations at the ecological level, but also impacts plant genome evolution. In this study, the first published flow cytometry estimate for *R. pentagyna* and a confirmation of the previously reported estimate for *R. lutea* were presented alongside the validation and comparison against the estimates via the exploitation of short-read sequence data k-mer analysis. However, some k-mer-based tools demonstrated consistency with flow cytometry estimates. Unfortunately, k-mer analysis remains problematic since its estimates fluctuate based on the tool parameter choices as well as coverage and quality of reads. When fresh material and enough resources are available, flow cytometry should be the preferable method for determining genome size, and kmer should be used solely to provide an approximate estimate. Furthermore, the substantial proportion of repeated elements identified in both species could imply that the expanded genome resulted from repetitive element amplification along with polyploidization. Based on our results and the rise in chromosome number, we hypothesize that *R. lutea* has a tetraploid genome or higher. More research is needed, however, to validate the ploidy type. The information acquired from this study should provide a basis for future phylogenetic and evolutionary studies, as well as the initiation of genome sequencing projects at the chromosome level.

## Figures and Tables

**Table 1:** C-value comparison (flowcytometry vs. k-mer) genome size and G0/G1 phases of the cell cycle in *Reseda lutea* and *Reseda pentagyna*. Genome size (mean ± standard deviation). The number of individuals analyzed for genome size (***n***).

	*n*	G0/G1 (%)	2C DNA Content (pg)	1C DNA Content (pg)	1C DNA Content (Mbp)	2C K-Mer (pg)	1C K-Mer (pg)	1C K-Mer (Mbp)
***Reseda lutea***	3	86.53 ± 2.20	1.91 ± 0.02	0.955 ± 0.01	973.11	1.78	0.89	867.7
***Reseda pentagyna***	3	80.96 ± 0.83	2.09 ± 0.029	1.045 ± 0.015	1026.9	1.84	0.92	896.3

* The external reference standard [*Solanum lycopersicum* (2C = 1.96 pg)] * c-values with the two methods are significantly different at *p* < 0.01 (one-sample *t*-test).

**Table 2:** Cytogenetical characteristics of *Reseda* species [64,65].

	Species	2C (pg)	Chromosome Number	Ploidy Level	Basic Chromosome Number	Section
**1**	***R. lutea***	2.06	24, 48	4-8	6	Resedastrum
**2**	***R. stricta***	2.86	24	4	6	
**3**	***R. lanceolata***	1.70	24	4	6	
**4**	***R. odorata***	0.96	12	2	6	Phyteuma
**5**	***R. phyteuma***	1.34	24	4	6	
**6**	***R. media***	2.09	12	2	6	
**7**	***R. undata***	1.22	20	4	5	Leucoreseda
**8**	***R. barrelieri***	1.68	20	4	5	
**9**	***R. suffruticosa***	0.92	20	4	5	
**10**	***R. alba***	1.45	40	8	5	
**11**	***R. luteola***	1.75	24	4	6	Luteola
**12**	***R. glauca***	2.11	28	4	7	Glaucoreseda
**13**	***R. complicata***	1.71	28	4	7	
**14**	***R. virgata***	1.44	28	4	7	
**15**	***R. gredensis***	2.63	28	4	7	

**Table 3:** Summary statistics for *Reseda lutea* and *Reseda pentagyna* genome sequences.

Sample ID	Fragment Length (bp)	Read Length (bp)	Total Reads	Clean Unmapped Reads ^a^	GC(%)	AT(%)	Q20(%)	Q30(%)
*Reseda lutea*	614	2 × 151	716,375,240	637,861,144	45.01	54.99	95.83	90.59
*Reseda pentagyna*	617	2 × 151	704,839,182	632,346,592	52.83	47.17	97.18	92.59

^a^ Clean reads: The number of reads that have survived after quality trimming and contamination removal.

**Table 4:** K-mer estimations of genome size (Mbp) utilizing raw (**R**) and quality processed (**P**) sequencing data for *Reseda lutea* and *Reseda pentagyna*.

***Reseda lutea***							
**Genome Estimation Software**	**K21**		**K31**		**K41**		**Average** **Processed data (SD)**
	**R**	**P**	**R**	**P**	**R**	**P**	
**General Formula**	845	851	860	868	876	884	867.7 (16.5)
**FindGSE**	864	876	972	988	1077	1078	980.7 (101.2)
**Covest-Repeat**	826	772	885	958	1123	1209	979.67 (219.3)
**Kmergenie**	391	401	471	483	542	559	447.7 (132.6)
**GenomeScope V1**	584	591	652	665	788	796	684 (103.8)
***Reseda pentagyna***							
**Genome Estimation Software**	**K21**		**K31**		**K41**		**Average** **Processed data (SD)**
	**R**	**P**	**R**	**P**	**R**	**P**	
**General Formula**	871	880	874	882	931	927	896.3 (26.6)
**FindGSE**	768	781	825	848	935	971	866.7 (96.4)
**Covest-Repeat**	817	825	1010	1067	1249	1318	1070 (246.51)
**Kmergenie**	484	486	582	591	611	614	552.3 (66.4)
**GenomeScope V1**	515	524	602	619	723	748	630.3 (112.4)

(**K21**) (**K31**) (**K41**) k-mer sizes; (**SD**) Standard Deviation.

**Table 5:** Genome properties of *Reseda lutea* and *Reseda pentagyna*.

Genome size Property *	*Reseda lutea*		*Reseda pentagyna*	
	min	max	min	max
**Homozygous (%)**	98.96	98.96	98.63	98.63
**Heterozygous (%)**	1.04	1.04	1.37	1.37
**Genome Haploid Length (bp)**	789,888,133	796,236,693	747,545,978	747,661,754
**Genome Repeat Length (bp)**	562,888,521	566,147,654	424,413,611	424,480,553
**Genome Unique Length (bp)**	226,999,612	230,089,039	323,132,368	323,181,201
**Model Fit (%)**	91.35	97.38	95.13	98.3
**Read Error Rate (%)**	0.06	0.06	0.12	0.12
**Repeats (%)**	71.26	71.1	56.77	56.77

* Estimated from processed reads by GenomeScope v1 with k = 41.

## Data Availability

The data which support the results of this study are available in GenBank at the NCBI (https://www.ncbi.nlm.nih.gov, accessed on 3 July 2021) under the BioProject accession PRJNA733338. NCBI accession numbers for all species in the molecular identification analysis are available in Figure 2.
